# Comparison of Serum IgG Antibody Test with Gastric Biopsy for the Detection of *Helicobacter Pylori* Infection among Egyptian Children

**DOI:** 10.3889/oamjms.2015.062

**Published:** 2015-06-03

**Authors:** Mones M. Abu Shady, Hanan A. Fathy, Alaa Ali, Essam M. Galal, Gihan A. Fathy, Hiba Sibaii

**Affiliations:** 1 *Department of Child Health, Medical Research Division, National Research Centre, Dokki, Giza, Egypt*; 2 *Health Radiation Research Department, National Center for Radiation Research and Technology, Atomic Energy Authority, Cairo, Egypt*; 3 *Department of Medical Physiology, National Research Centre, Cairo, Egypt*

**Keywords:** *Helicobacter pylori*, serologic test, children, Egypt

## Abstract

**BACKGROUND:**

In developing countries, *Helicobacter pylori* (*H. pylori*) infection is mainly acquired during childhood and may be a predisposing factor for peptic ulcer or gastric cancer later in life. Noninvasive diagnostic tools are particularly useful in children for screening tests and epidemiological studies. Data on serologic testing of children are lacking. Accurate noninvasive tests for diagnosing Helicobacter pylori infection in children are strongly required.

**AIM:**

The aim of this study was to evaluate the performance of a serological test (serum IgG antibody for *H. pylori*) in Egyptian children with recurrent abdominal pain necessitating endoscopy.

**SUBJECTS AND METHODS:**

One hundred children, referred to the endoscopy unit at Mansoura University. Upper endoscopy was done for each with rapid urease test (RUT) and histological examination as the gold standard test for detection of *H. pylori* infection. Serum samples were collected for detecting IgG for *H. pylori* infection.

**RESULTS:**

The mean age of the subjects included in the study was 7.23 ± 1.94 year. Serological test (IgG to *H. pylori*) was positive in 60% of all cases. A highly significant association between the standard test and the serological test at a cutoff > 10 U/ml at p = 0.001 were detected for the diagnosis of *H. pylori* infection. The sensitivity, specificity, positive likelihood ratio, and negative likelihood ratio for the IgG antibody a cutoff > 10 U/ml, were 96.5%, 93%, 13.83, 0.038 respectively.

**CONCLUSION:**

Serum IgG antibody to *H. pylori* infection has a high diagnostic value and can be considered as a suitable and reliable noninvasive test for detection of *H. pylori* infection.

## Introduction

At least half of the world’s population is estimated to be infected with *H. pylori*. However, the prevalence of this infection varies widely across both geographic regions and ethnic groups. Overall, rates of *H. pylori* infection are markedly higher in developing countries compared to developed countries. For example, prevalence rates that approach or even exceed 90% have been reported in multiple studies conducted in Bangladesh, Egypt, Russia, Siberia, and Africa. In contrast, prevalence rates are much lower in developed regions, including the United States (6.8–79%), Europe (7.3–70%), and Australia (15.5–23%) [1-3]. Most infections are probably acquired in childhood, mainly *via* oral-oral or fecal-oral routes [4].

*Helicobacter pylorus (H. pylori) causes* gastrointestinal diseases such as gastritis and peptic ulcer in adults and children. In addition, previous reports have linked *H. pylori* infection with gastric cancer, mucosa-associated lymphoid tissue (MALT) lymphoma, iron deficiency anemia and thrombocytopenic purpura in children [5-7].

Diagnostic methods for *H. pylori* infection are usually classified as invasive and noninvasive. The invasive tests including histology, urease tests and culture, require upper gastrointestinal endoscopy for obtaining the diagnostic sample. On the other hand, non-invasive methods include the urea breath test, serology and stool antigen test. Bacterial culture from the gastric biopsy is the gold standard technique, and is recommended for antibiotic susceptibility test. Serology is used for initial screening and the stool antigen test is particularly used when the urea breath test is not available [8]. To define the value or usefulness of a diagnostic test, each test has to be compared to a gold standard [9]. There are few data on serologic tests for children, and thus it remains unclear whether the serology cutoffs used for adults are applicable to children.

The aim of this study was to determine the accuracy of the noninvasive serologic test in comparison with the invasive gold standard (endoscopy with biopsy analyses) for the diagnosis of *H. pylori* in Egyptian children with different upper gastrointestinal disorder.

## Material and Methods

One hundred children (age range 4-10 years), referred to the endoscopy unit at Mansoura University Children Hospital for upper gastrointestinal disorder, were recruited in the present study. Informed consent was obtained from the parents of the children. The study was approved by the Ethical Committee of National Research Centre.

Patients were excluded from the study if they had received treatment with antibiotics, proton pump inhibitors, and H2 receptor antagonists within the last four weeks. Patients with previous gastric surgery, long-term use of corticosteroid and immunosuppressant, and history of bleeding or active gastrointestinal bleeding were also excluded from the study.

During upper endoscopy (Olympus GIF P 230; Olympus Optical Co., Tokyo, Japan), three gastric biopsies (two taken within 3 cm from the pylorus and one from the corpus) were taken. One biopsy was used for rapid urease test (RUT) (Dio-Helico, Diomed), and the remaining two biopsies were used for histological examination (Hematoxiline and Eosin staining) for H. pylori infection. A rapid urease test result was obtained by adding a biopsy specimen to a urea broth (NaCl, KH_2_PO_4_, and NaOH); the result of the test was considered positive if there was a change of urea broth color from yellow-gold to pink-red due to an increase in pH induced by *H. pylori* [10].

Serum samples were stored at −20°C until the laboratory assay was performed. Serum antibodies (IgG) to *H. pylori* were examined using a microplate enzyme immunoassay (EIA) and an antibody determination kit (E-Plate Eiken *H. pylori* antibody, Eiken Chemical Co., Ltd., Tokyo, Japan). All samples were analyzed according to the manufacturer’s instructions, and the cutoff point was set at 10 U/ml. All assays were performed by experimenters blinded to the clinical status of the patients.

The gold standard for the presence of *H. pylori* infection was defined as both the histological examination and rapid urease test being positive. The absence of *H. pylori* infection required both tests to be negative.

### Statistical Analysis

Statistical analysis was carried out using the statistical package for social sciences, version 16 for windows (SPSS Inc., USA). Continuous data were expressed as mean± SD, while Categorical data were expressed as frequencies and percentages, and were analyzed with the two-tailed chi-square test. The chi-square(χ^2^) test, odds ratio (OR) and 95% confidence interval (CI) were used to evaluate the association between serum IgG at a cutoff 10 U/ml and the gold standard (RUT and histological examination) for detection of *H. pylori* infection. To assess the criterion validity of the serologic test, sensitivities, specificities, positive likelihood ratios, and negative likelihood ratios were estimated relative to the gold standard, across all possible cutoff values for the serologic test. Receiver operating characteristics (ROC) analysis was also conducted using the gold standard to assess the performance of serum IgG in detection of *H. pylori* infection. The 95% CI of the area under the ROC curve (AUC) was calculated. *P* value < 0.05 was considered statistically significant.

## Results

Of the one hundred subjects included in the study 57 were male and 43 were female. Their age ranged from 4-10 years with a mean age 7.23 ± 1.94 year. Standard test were positive in 57% of all cases and negative in 43%. Serological test (IgG to *H. pylori*) was positive in 60% of all cases and negative in 40%.

Table 1 showed a highly significant association between the standard test and the serological test at a cutoff > 10 U/ml (Chi-square = 80.6, Odds ratio = 5.904, 95% Confidence interval = 4.069-7.739, and p < 0.001).

**Table 1:** Association between serological test and the standard test

		Standard test		Total	χ^2^	OR	95% CI	p
	
		Positive	Negative			
Serology test (IgG> 10 U/ml)	Positive	55	3	58	80.6	5.904	4.069-7.739	0.000*

	Negative	2	40	42				

Total		57	43	100				

P < 0.005 is significant; χ^2^ = Chi-square; OR = Odds ratio; CI = Confidence interval.

As shown in Table 2, when the cutoff point of IgG antibody to *H. pylori* recommended by the manufacturer was used, the sensitivity, specificity, positive likelihood ratio, and negative likelihood ratio were 96.5%, 93%, 13.83, 0.038, respectively. Sensitivity and specificity at different cutoff values of IgG antibodies to *H. pylori* were shown in the Table 2.

**Table 2:** Sensitivity and specificity of anti-*H. pylori* IgG antibody test for Egyptian children, by cutoff point

Cutoff (U/ml)	Sensitivity (95% CI)	Specificity (95% CI)	LR+ (95% CI)	LR- (95% CI)
3	99.1 [0.922-0.999]	98.9 [0.899-0.999]	86.243 [5.481-135.11]	0.009 [0.001-0.139]
4	99.1 [0.922-0.999]	85.3 [0.622-0.719]	80,136 [0.969-1.115]	0.187 [0.009-4.042]
5	99.1 [0.922-0.999]	87.9 [0.722-0.819]	54.345 [1.14-1.659]	0.031 [0.002-0.513]
6	98.2 [0.907-0.997]	86.3 [0.625-0.711]	1.837 [1.387-2.432]	0.038 [0.005-0.27]
7-9	96.5 [0.881-0.99]	93 [0.814-0.976]	13.83 [4.638-41.239]	0.038 [0.01-0.148]
10	96.5 [0.881-0.99]	93 [0.814-0.976]	13.83 [4.638-41.239]	0.038 [0.01-0.148]
11	82.5 [0.706-0.902]	95.3 [0.845-0.987]	17.728 [4.557-68.974]	0.184 [0.104-0.324]

LR = likelihood ratio.

**Figure 1:**
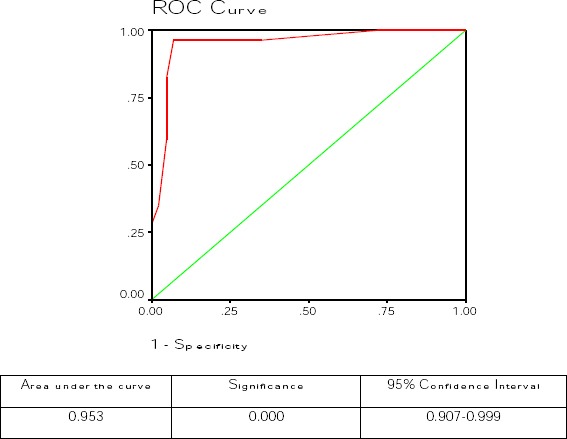
*Receiver operating characteristics (ROC) curve for anti- H. pylori IgG antibody test, with the histological examination and RUT as the gold standard*.

Figure 1 showed receiver operating characteristics (ROC) curve for anti-*H. pylori* IgG antibody test, with the histological examination and RUT as the gold standard. The area under the curve (AUC) for the anti-*H. pylori* IgG antibody test was 0.953 and 95% Confidence interval 0.907-0.999.

## Discussion

*H. pylori* is acquired in childhood and survives in the human stomach. Noninvasive testing for *H. pylori* has been strongly recommended as it is less expensive and more patient-friendly than invasive testing that requires endoscopy [11]. Serology was one of the first methods used for diagnosis of *H. pylori* infection. Detection of antibodies is useful for detecting past or present exposure [12]. In fact, a limitation of serology tests is the failure to distinguish between past and current *H. pylori* infection [13].

Serological tests have several advantages, namely they are non-invasive and they do not produce false negative results in patients receiving treatment (proton pump inhibitors and antibiotics) or presenting acute bleeding [14]. The success of a serology test depends on the use of antigens that are present in *H. pylori* strains from a given population. Moreover, kits developed using *H. pylori* strains from the west are not suitable for detecting *H. pylori* infection in the East [15]. The use of high-molecular-weight cell associated antigens that are conserved in *H. pylori* strains overcomes this limitation [16].

In the present study, a highly significant association was detected between the standard test for *H. pylori* (histological examination and RUT) and serological test (IgG antibody for *H. pylori*) for detection of *H. pylori* infection. A sensitivity and specificity of 96.5% and 93% were detected using a cutoff level of IgG of 10 U/ml with AUC of 0.953 with significance of <0.001 at the ROC curve.

Previous studies reported debate regarding the use of serologic tests for detection of *H. pylori* infection in children [18, 19]. Okuda et al., [18] studied 157 children for comparing antibodies to *H. pylori* (IgG and IgA enzyme linked immunosorbent assay (ELISA)) with *H. pylori* stool antigen (HpSA) enzyme immunoassay. They concluded that an immature immune response or tolerance to *H. pylori* exists in childhood and sero diagnosis of *H. pylori* infection is less useful in children aged below 10. Frenck et al., [19] studied children between 2 and 17 years of age, evaluated at the Cairo University School of Medicine Pediatric Gastroenterology Clinic who were already scheduled for upper endoscopy. Rapid urease, histology, and culture were done as invasive tests. Urea breath test performed. Stool and serum samples were tested for the presence of *H. pylori* by using commercially available enzyme-linked immunosorbent assay-based technology. They concluded that urea breath test and stool enzyme-linked immunosorbent assay kit had the highest sensitivity and specificity (sensitivity and specificity: 98 and 89 [urea breath test] and 94 and 81 [HpSA], respectively). In contrast to the present study, the serologic kit in their results had an unacceptably low sensitivity (50%).

In agreement with the results of the current study, a 2008 meta-analysis of 42 studies of children showed a sensitivity of 79.2% (95% CI, 77.3–81.0) and a specificity of 92.4% (95% CI, 91.6–93.3) for a serologic IgG antibody test [19]. In 2014, Ueda et al., [20] studied the performance of the E-plate anti-*H. pylori* IgG antibody test and it was found to be comparable to that of the stool antigen test. In concordance with the result of the present study, they found sensitivity, specificity, AUC of IgG (cutoff value 10 U/ml) of 91.18%, 97.44%, o.96 respectively. They concluded that IgG antibody to *H. pylori* might be useful in epidemiologic studies involving large numbers of participants. In agreement with the present study, Alam El-Din et al., [21] concluded that diagnosis of *H. pylori* infection by noninvasive methods, including the serum antibody test, revealed a sensitivity and positive predictive value of 88.9% and 94.2%, respectively.

A previous study released by (HO and Marshall,) [22] recommended that, in studies using serologic tests, researchers should reexamine the test results and determine if it is necessary to adopt specialized cutoff points in children. Accordingly, the performance of the serologic test was evaluated in the present study by using various cutoff points and it was found that cutoff points in the range of 7 to9 U/ml yielded optimal sensitivity, specificity, and positive likelihood ratio.

The present study has several limitations. First, because only a subset of children for whom both blood and endoscopy was available were included in this study. The results need to be replicated in other, larger samples of consecutive patients. Second, IgG antibodies can be detected approximately 3 weeks after *H. pylori* infection. Therefore, the latent period between *H. pylori* infection and antibody production may be a source of misclassification. Finally, information, such as number of siblings and birth order, have not been collected that may be related to transmission of *H. pylori* infection among children.

In conclusion, IgG antibody for the detection of *H. pylori* infection seems to be a good alternative for invasive diagnostic tests such as urea breath test. IgG antibody to *H. pylori* has a high diagnostic value and can be considered as a suitable and reliable noninvasive test for detection of *H. pylori* infection. The choice of test kit depends on the sensitivity and specificity in each region and the circumstances of each patient. Further studies are needed to explore genetic differences among populations and their effects on immune responses.
